# Stakeholder perspectives on factors that influence global prioritization for MNH in humanitarian settings

**DOI:** 10.3389/fgwh.2024.1364603

**Published:** 2024-08-26

**Authors:** Alicia Adler, Naoko Kozuki, Mamothena Mothupi

**Affiliations:** ^1^ Airbel Impact Lab, International Rescue Committee, New York, NY, United States; ^2^ Airbel Impact Lab, International Rescue Committee, Washington, DC, United States; ^3^ Airbel Impact Lab, International Rescue Committee, Nairobi, Kenya

**Keywords:** maternal and newborn health, humanitarian and fragile setting, priority setting, health policy prioritization, global health agenda

## Abstract

**Background:**

Maternal and newborn mortality rates are disproportionately high in crisis and conflict-affected countries. This study aims to understand factors influencing how MNH in humanitarian and fragile settings (HFS) is prioritized on the global health agenda during the Sustainable Development Goal (SDG) era. This includes examining the policies and processes driving agenda setting and decision-making, as well as the perceptions of global actors. It further reflects on the role of global milestones, reports, convenings, and high-level champions, based on the premise that global prioritization leads to increased attention and resource allocation, ultimately contributing to improved outcomes for mothers and newborns in crisis-affected areas.

**Methods:**

A qualitative study conducted from April 2022 to June 2023, employing a desk review and 23 semi-structured key informant interviews with global actors from donor agencies, implementing organizations, research institutes, United Nations agencies, professional associations, and coalitions, predominantly based in the Global North. Data were analyzed using inductive thematic analysis and the research was guided by the Walt and Gibson Health Policy Triangle framework.

**Results:**

Participants believe that global agenda-setting and investment decisions for MNH are primarily driven by UN agencies, donors, and implementing organizations at the global level. Although the Millennium Development Goal era successfully prioritized MNH, this focus has diminished during the SDGs, especially for HFS. Identified barriers include the complexity of reducing mortality rates in these contexts, limited political will, MNH investment fatigue, and a preference for quick wins. Fragmentation between humanitarian and development sectors and unclear mandates in protracted crises also hinder progress. Without enhanced global advocacy, accountability, and targeted investments in HFS, respondents deem global MNH targets unattainable.

**Conclusions:**

While waning donor interest and the siloing of HFS in global MNH decision-making pose challenges, targeted actions to address these barriers may include designating quotas for humanitarian actors in global MNH convenings, developing shared messages that convey common interests, and adopting an equity lens. Prioritizing MNH in HFS on the global agenda demands sustained commitment to ensure these settings are not an afterthought through dedicated advocacy and accountability, high-level political engagements, global milestones, and by leveraging opportunities to capture mainstream interest. Failing to shift global priorities will result in continued stagnation and worsening MNH outcomes across HFS.

## 1 Introduction

Maternal and newborn health (MNH) encompasses the health of women during pregnancy, childbirth, and the postpartum period, as well as the health of newborns during their first month of life (1). United Nations (UN) data published in 2023 shows that progress to reduce maternal deaths is stagnating, with countries affected by humanitarian crises lagging furthest behind, contributing to 58% of global maternal deaths, 38% of newborn deaths, and 36% of stillbirths (2, 3). South Sudan, for example, is among the world's most fragile countries and reports 1,223 maternal deaths per 100,000 births and 39 newborn deaths per 1,000 live births (4–6).

To mobilize efforts around MNH, the UN launched the Global Strategy for Women's, Children's and Adolescents' Health to serve as a global roadmap for improving the health and well-being of women, children, and adolescents, and ending preventable deaths by 2030 (7). While this strategy recognizes that ending preventable deaths hinges on a commitment to equity through targeted efforts aimed at marginalized populations, hard-to-reach communities, and humanitarian and fragile contexts, improving MNH outcomes in humanitarian and fragile settings (HFS) is complicated by conflict, political instability, natural disaster, and other crises (8).

These settings are often characterized by heightened vulnerabilities, including disrupted health systems, displacement, and limited access to essential services. The complex socio-political environments in fragile settings often challenge effective government intervention and complicate the coordination of development and humanitarian actors who support health system strengthening and the provision of essential health services, including MNH care (9).

Given the disproportionate burden of maternal and newborn mortality across HFS, there is an urgent need to prioritize MNH in HFS on the global health agenda. In this study, global prioritization refers to the degree to which MNH in HFS gains visibility on the global health agenda, contributing to the allocation of technical, financial, and human resources needed to accelerate progress and ultimately improve outcomes for mothers and newborns in crisis-affected areas (10).

Although research has explored the prioritization of MNH during the Millennium Development Goal (MDG) era and the political economy of humanitarian aid, there is a gap in understanding how MNH in HFS has been prioritized on the global health agenda since the shift to the Sustainable Development Goals (SDGs) post-2015 (11–14).

Aligned with the Shiffman and Smith framework for global political priority, our research explores various aspects of prioritization including political milestones, thematic inclusion in global report and forums, advocacy by high-level champions, and more. While research underscores that achieving global political priority alone is insufficient, it remains a critical enabling factor for effectively addressing MNH challenges (10, 11).

## 2 Methods and materials

This study examines the systems, processes, and perceptions of global actors—namely individuals working at the headquarter offices of donor agencies, international non-governmental organizations (INGOs), UN agencies, and academic institutions—to better understand what influences the prioritization of MNH in HFS on the global health agenda.

### 2.1 Study design

This study uses a single-case, holistic, descriptive case study approach to examine the prioritization of MNH in HFS on the global level during the SDG era (12). We chose a case study design to capture stakeholders' perspectives on global MNH prioritization and conducted a desk review of literature on global MNH action in recent decades.

### 2.2 Conceptual framework

**Figure 1:**
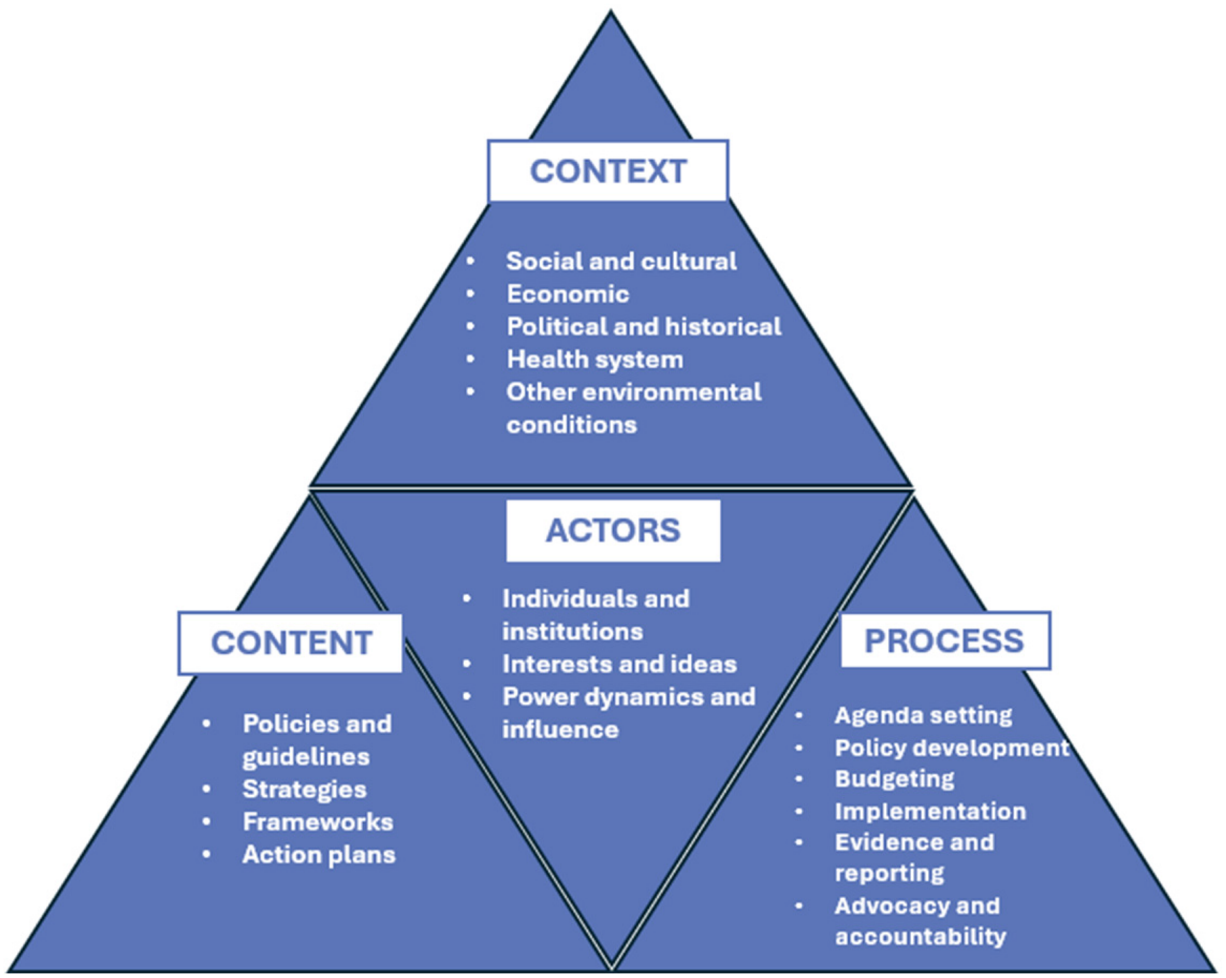
Walt and Gilson, Health Policy Triangle Framework (1994).

The study leverages Walt and Gilson's Health Policy Triangle (HPT) framework (shown in Figure 1) to explore perceptions among global actors, the content of policies and strategies, the context of shifting global priorities, and the humanitarian development processes that influence MNH program investment and visibility over time (13). Each pillar of Walt and Gibson's framework, further explained in Box 1, is interconnected and influences one another. The framework was used to design data collection tools, support analysis and interpretations, and structure the study findings. During analysis, the Shiffman and Smith framework on political priority was used to aid interpretations and make recommendations (10). In particular, the Shiffman and Smith framework unpacks political aspects of prioritization by highlighting the global policy community, the influence of strong public leaders and champions, opportune policy windows, civil society mobilization, the resonance of external narratives, and the role of global governance structures as platforms for collective action and accountability.

Box 1 **Content explored through the Walt and Gilson framework pillars.**

**Context**: Examined historical milestones marking progress and challenges within the MNH sector.

**Content**: Assessed relevant global MNH strategies and guidelines to determine how humanitarian and fragile settings are included.

**Actors:** Considered different MNH stakeholders at the global level including the diverse interests that impact priority-setting and decision making.

**Processes:** Studied global level processes tied to governance, financing, and accountability for global mortality reduction targets while comparing development vs. humanitarian coordination and prioritization efforts.

### 2.3 Setting

The study is focused on the global health policy landscape for MNH in HFS, including actors working at the global level. In this case, the global level refers to the activities and efforts that transcend national borders and involve coordination, collaboration, and policymaking at an international level to address MNH challenges across contexts. Guided by the WHO's definition of health policy as “decisions, plans, and actions undertaken to achieve specific healthcare goals within a society” this involves examining the broader global health policy context including the systems, processes, and perceptions of global actors influencing the prioritization of MNH in HFS on the global health agenda (14).

### 2.4 Data collection

The initial phase of the study involved a desk review of existing evidence on the global policy landscape for MNH in HFS. This review helped to identify evidence gaps, which, along with the Walt and Gibson framework, guided the subsequent data collection through key informant interviews.

#### 2.4.1 Desk review

The desk review was done iteratively using search criteria on scholarly databases and snowball searching of reference lists and organizational websites. The review included peer reviewed and grey literature, focused on global MNH or Sexual and Reproductive Health and Rights (SRHR) strategies and commitments, global guidelines, publicly available advocacy and donor reports, webpages of relevant implementing organizations, working groups, and consortiums, academic studies, strategic plans (UN, NGOs, donors), and media articles.

The search strategy included using a range of search terms (“political economy” AND “maternal and newborn health” OR “MNH”; “MNH AND humanitarian”, “MNH and fragile settings”, “global health AND agenda setting”; “MNH AND prioritization”; “geopolitics AND humanitarian”; “health AND global financing, humanitarian system”) adapted with slight variations to the following databases: Google Scholar, PubMed, and Scopus. Similar key terms were used in Google and websites for the Partnership for Maternal, Newborn and Child Health (PMNCH), United Nations Population Fund (UNFPA), United Nations Children's Fund (UNICEF), the World Health Organization (WHO), and the Inter-Agency Working Group on Reproductive Health in Crises (IAWG). Selection criteria prioritized content from reputable sources, published in English, that provided insights into global MNH or SRHR strategies, commitments, guidelines, advocacy efforts, donor perspectives, and academic studies pertinent to the study's focus on MNH in humanitarian and fragile settings. A total of 80 sources are referenced throughout the paper (including in Supplementary 1). The formal analysis informing the study findings includes 10 peer-reviewed articles and 15 pieces of grey literature, mainly from UN and NGO reports.

#### 2.4.2 Key informant interviews (KII)

We conducted a stakeholder mapping exercise to identify key informants who work transnationally and are actively engaged in global policymaking, practice, and financing for MNH in stable contexts and across HFS. This includes implementing organizations, academic institutions, public and private donors, bilateral and multilateral agencies, among others. Initially, we determined the relevant categories of stakeholders based on our research objectives and questions. We then mapped individuals within each category by reviewing references in literature and consulting representatives from various global MNH in HFS working groups and coalitions. Additionally, we employed snowball sampling by asking key informants to recommend others who could provide valuable insights. 20 semi-structure KIIs were conducted between June 2022 and October 2022 with three additional interviews conducted in June 2023 to capture insights from the International MNH Conference held in May 2023. KIIs included representatives from donor agencies (*n* = 6), NGOs (*n* = 6), researchers/research institutes (*n* = 4), UN agencies (*n* = 5), and professional associations (*n* = 2). The sample predominantly consisted of individuals based in the Global North (22 out of 23 people), specifically in the US, UK, and Switzerland (Geneva). While participant selection was not purposively exclusive of Global South-based stakeholders, our sample reflects the enduring power and influence the Global North holds over the global health agenda (15). The total sample of the study was also influenced by time constraints and the availability of respondents during the data collection period.

Interviews focused on better understanding the systems and processes shaping global policy making and financing for MNH in HFS, as well as the perceptions that influence the current level of prioritization on the global health agenda. We adapted the interview guides for different participant categories (donor, INGO, researcher/academia, UN) to align questions with their experience and expertise. All interviews were conducted by the first author via Microsoft Teams and Zoom in English and lasted approximately one hour. Verbal informed consent was obtained, and interviews were audio recorded with automatic transcription enabled and verified for accuracy.

### 2.5 Data analysis

The data analysis followed an inductive, thematic approach, utilizing Walt and Gibson's triangle framework to organize the findings. Data from the desk review was extracted into a Microsoft Word document organized according to the framework. Each source was reviewed, and findings related to each of the HPT domains were summarized, with sub-themes identified based on reoccurring patterns across the literature. Data from the KIIs was coded twice: at the first level, we reviewed and re-reviewed transcripts and created descriptive codes related to the overarching study framework. This was followed by a second level pattern coding applied across the initial codes, to identify sub-themes related to the framework domains of content, process, context, and actors. Data underwent further reduction into themes during the synthesis of desk review and interview findings and were ultimately organized according to the Walt and Gibson framework. The thematic analysis relied on reoccurring inductive codes to assess relative data richness across themes and subthemes.

### 2.6 Rigor and trustworthiness

To ensure rigor and trustworthiness, we relied on two data sources (desk review and KIIs), triangulation, and validation of results. To minimize researcher bias and enhance the dependability of the findings, debriefing sessions were held with the co-authors after every three to five KIIs. During design and analysis, researchers discussed and agreed on common understanding and application of the conceptual framework used to guide the study. Researchers held periodic meetings to discuss emerging themes and redraft themes and sub-themes by consensus. Data collection and analysis were conducted iteratively to ensure thorough exploration of themes. Saturation was achieved when no new information or themes emerged from the key informant interviews (KIIs) or desk review (16). Following analysis, the findings were presented to individuals, including members of the IAWG MNH sub-working group, with experience engaging in the spaces being studied, to validate our results.

### 2.7 Reflexivity

This study was conducted by three global health researchers working in an INGO specializing in humanitarian and fragile settings. The first author has more than ten years of experience in policy and advocacy, with several years specifically focused on MNH for development and humanitarian organizations at global and country levels. The second author is a senior researcher with vast experience leading research programs in SRHR and MNH in academia and the NGO sector at the global level and within LMIC contexts. The last author is based in the Global South and has worked as a public health, health policy and systems researcher over the past 12 years within government, academia, and NGOs. The diversity of author experiences informed the design and methodology of the study, access to participants, as well as the perspectives and interpretations of this study.

### 2.8 Ethics

Respondents voluntarily participated and provided informed consent for recorded interviews. Data analysis was anonymized to protect participant identities. This research received ethical approval from the International Rescue Committee Institutional Review Board (USA).

## 3 Findings

The findings are organized according to context, content, processes, and actors, aligning with the Walt and Gibson framework.

### 3.1 The context of MNH prioritization on the global health agenda

To understand the global policy landscape for MNH in HFS, it is important to review the historical context of MNH in development and humanitarian settings, and to analyze the factors and milestones that shaped MNH progress during the MDG and SDG eras.

#### 3.1.1 MNH milestones and momentum

Securing recognition for MNH on the global agenda took decades, with periods of accelerated progress triggered by milestone publications, events, and new initiatives (Supplementary Figure S1). Study respondents felt the introduction of the UN MDGs and the inclusion of maternal health in MDG5 was pivotal in creating favorable conditions for advocates to catalyze global action, investment, and learning.

*Having been working in the field prior to the establishment of the MDGs, it was very interesting to see how the MDGs motivated a lot of people to make sure they were working towards the priorities as jointly outlined and that they were being measured, tracked and shared publicly. (Respondent 14, NGO)*

While the SDG agenda was developed through extensive consultation, respondents contend that MNH has been overshadowed by its broader focus. One UN representative said:

*Under the MDGs, maternal health had its own goal and newborn health had the Every Newborn Action Plan so there was a lot of momentum. But I think as we transitioned to the SDG era with the focus on universal health coverage and primary healthcare, there are so many things to focus on and I think we're really losing the focus on MNH as its own issue. (Respondent 8, UN)* ^1^

A report published by the United Nations Maternal Mortality Estimation Inter-Agency Group in 2023 indicates that between 2000 and 2020, progress to reduce maternal mortality stalled in 133 countries, with 17 countries experiencing an increase in maternal death, leaving the world severely off track to meet SDG targets related to MNH (2). Some respondents believe momentum around MNH has stalled in part due to the lack of global attention and high-level leadership needed to drive action and accountability toward global MNH targets.

Global MNH targets (90/90/80/80) announced in May 2023 set a new, unified, and measurable focus with goals such as 90% of pregnant women receive at least four antenatal care contacts, 90% of births attended by skilled health workers, 80% of new moms and babies receive postnatal care within two days of birth, and 80% of country districts can access to emergency obstetric services and small and sick newborn care (17). While respondents acknowledge the importance of having shared goals to align and mobilize stakeholders, some argue these targets will be unattainable in HFS and instead see it as an opportunity to analyze data, address equity gaps, and bring attention to overlooked regions.

#### 3.1.2 Resource allocation

Several mechanisms such as Countdown 2030 (formerly Countdown 2015), Muskoka RMNCH, and the Institute for Health Metrics and Evaluation, track RMNCH funding, though year-on-year trends differ based on the approach (18). Throughout most of the MDG era, global aid for RMNCH increased, yet when looking at the proportion of funding allocated to MNH specifically, a different narrative emerges with MNH budget lines often receiving less funding than those designated for reproductive health (especially HIV/AIDS) and child health (18, 19). A closer look at development assistance for RMNCH in 25 conflicted-affected settings shows similar trends from 2003 to 2017 with reproductive health receiving the largest share (50%), followed by child health (30%), MNH (18%) and adolescent health (2%) (20).

The transition to the SDGs saw global aid for RMNCH consistently declining (21, 22). The pandemic worsened this, also leading to reduced domestic financing for MNH across the many of fragile and conflict-affected countries (17). Respondents view the MDG funding growth as a reflection of donor interest in MNH and see more recent cuts as indicative of changing donor priorities.

#### 3.1.3 The influence of political events on the global prioritization of MNH in HFS

Global events, including elections and world forums, create policy windows that foster political will and momentum. Research shows that elections in high-income countries, for example, impact development and humanitarian assistance priorities with new administrations often setting different agendas, appointing leaders to development and foreign assistance agencies, and allocating budgets accordingly (23).

Previous research shows that political decisions, like the US Government's reinstatement of the Global Gag Rule in 2017, significantly impacted funding to SRHR and MNH programs. The decision forced some organizations to “choose between U.S. funding and comprehensive reproductive healthcare” contributing to clinic closures, reduced services, and an increase in unintended pregnancies and unsafe abortions, which account for approximately 13% of maternal deaths (24, 25).

Other geopolitical developments impact MNH investments, especially in regions where high-income countries have political, financial, or security related interests (26). Respondents highlighted that policymakers and donors from high-income countries strive to align foreign aid with interests of their home country, often responding to public opinion and pressure driven by media coverage (27). Almost all interviewees' cited Ukraine as an example of how geopolitics can shape media attention, public interest, and funding allocations with aid redirected from other contexts to respond to the ongoing crisis. With only so much humanitarian assistance to distribute and crises now lasting decades in many contexts, respondents indicated that places with the highest burden of maternal and newborn mortality often struggle to compete for funding and attention.

### 3.2 The inclusion of humanitarian settings in global MNH content

#### 3.2.1 Including HFS in global MNH guidelines and reports

Numerous global guidelines and reports are designed to address the prevention and management of maternal and newborn mortality and stillbirths. A table in Supplementary 1 outlines a non-comprehensive list of guidelines and reports relevant to the MNH sector including comments on if and how the document recognizes HFS. While many of these documents acknowledge the high burden of maternal and newborn mortality in HFS, respondents believed they are intended to be globally applicable, often without implementation guidelines for these contexts. Respondents further noted that humanitarian considerations are infrequently discussed during the development of normative guidance, with decision makers expecting national governments to adapt them. This approach was deemed problematic in many HFS where respondents said governments are perceived to be weaker and less likely to contextualize and operationalize such guidelines.

The WHO typically leads the development of MNH guidelines and although some civil society organizations and researchers participate as observers, respondents flagged that the inclusion of HFS in these discussions depends on stakeholders representation (28). Even when included, many said HFS are frequently an afterthought and often included as a case study or a reference to the need for adaptation to meet their unique circumstances.

Sector websites show that several documents do focus on MNH in HFS, namely the Inter-Agency Field Manual for Reproductive Health in Refugee Settings, Newborn Health in Humanitarian Settings: Field Guide, the Roadmap to Accelerate Progress for Every Newborn in Humanitarian Settings 2020–2024, and the Minimum Initial Service Package (29–32).

#### 3.2.2 Barriers to the inclusion of HFS on the global agenda

Respondents cite language as a barrier with humanitarian and development actors using different terminology, jargon, and acronyms, making MNH coordination challenging. There was a lack of consensus among respondents concerning the definition of HFS. Some respondents noted that the term “humanitarian” is often narrowly associated with acute emergencies and conflict rather than the broad spectrum of situations it is intended to encompass. They explained that this narrow perception can foster negative associations and create stigma surrounding MNH content labeled for HFS, potentially rendering it “irrelevant” to stakeholders focused on more stable contexts.

Another barrier identified by respondents was a belief that because every humanitarian crisis is unique, recommendations for operationalizing MNH interventions in one crisis are not readily applicable to others. This notion limits the perception of transferability of best practices and impedes the development of guidelines for MNH in humanitarian settings. A donor respondent explained:

*I think one of the big questions that we haven't answered is what are the parallels [between crises]? If you do research in a refugee camp in the DRC, to what extent does it hold to a typhoon in the Philippines? I don't think we know. I don't think we can say that there are or are not interchangeable but one of the things that always strikes me when I am in the immediate aftermath of a disaster or in a prolonged crisis, is the extent to which life goes on and people still need access to regular services] and I think it's very hard to intellectually grasp because you see these images of Haiti after the earthquake or, war-torn Somalia. (Respondent 1, donor)*

Respondents emphasized that interventions and standards should remain the same regardless of the context, but the difference is in how services are delivered.

### 3.3 Actors and institutions who influence the prioritization of MNH in humanitarian settings on the global health agenda

A multitude of global actors work on MNH in development and humanitarian settings. This includes UN agencies (e.g., UNICEF, UNFPA UNHCR, WHO), multi-laterals, implementing organizations like INGOs, researchers including academic institutions, professional associations, and numerous global networks, coalitions, and initiatives. The following section explores the influence, interests, and ideas of these actors in shaping the global prioritization of MNH in HFS.

#### 3.3.1 The power and influence of global actors

The 2016 Grand Bargain Commitments aimed to empower local actors in decision-making, agenda-setting, program implementation, and financial management (33). Despite this, many respondents assert that financing, decision-making, and agenda-setting power for MNH primarily remains at the global level, especially with high-income government donors.

Research shows that these dynamics impact not only what is funded, but also who is funded and where that funding goes geographically (34). In donor-dependent HFS, respondents claimed that decisions made by donors at headquarter offices have a cascading impact on country-level policies, programs, and research and national governments sometimes feel compelled to shift their priorities to secure funding aligned with donor interests. While a few respondents remarked on improvements in funding to local organizations, donors remain reluctant to relinquish power and prefer to invest in implementing organizations from the Global North as highlighted by this donor:

*I think we as donors are quite influential and honestly, I'm not sure we're ready to give that up yet. I know there's a lot of rhetoric around localizing aid, but I don't think we're ready to. We're quite happy to be influential on those funding mechanisms [humanitarian and health pooled funds] and in some cases where we feel we're not able to be influential and things are happening in a way that we don't want, we'll look at it and decide if we need to go with a more direct [funding] instrument where we've got control. (Respondent 5, donor)*

While respondents agree that national ministries of health should have the authority to make health decisions within their countries, they argue that there is a perception among many that this is more challenging in HFS where governments and health systems are strained. Consequently, respondents believe some donors may be hesitant to invest in these contexts.

Respondents further emphasized that power and influence over setting the global MNH agenda extend beyond donors with INGOs, networks, and global initiatives like Ending Preventable Maternal Mortality (EPMM), ENAP, IAWG, and PMNCH playing important roles in advancing MNH investment, coordination, information sharing, technical guidance, and expertise. There was recognition among most respondents that these entities are primarily composed of Global North institutions and individuals, with minimal representation from the countries being discussed.

Generally, respondents accepted this based on the different mandates and bandwidth of global level and national actors with one NGO representative stating “I still think that there's a notion of [global actors playing the role of] convening and curating. I think that is an appropriate role for global players to play as long as they're doing it in a way that engages multiple country actors”, (Respondent 14, NGO). Another individual indicated that “it's just not fair to the people in the field to be pulled in [to global engagements] because that's not their remit. If you're working in South Sudan, you have enough to do right there. You don't need to be helping IAWG be inclusive and diverse”. (Respondent 15, NGO)

Some respondents believe that COVID-19 helped shift to more online interactions, potentially “democratizing participation” in global MNH dialogues but there needs to be more meaningful engagement of diverse actors including from HFS. One individual expanded:

*Often there would be a global consultation of 30 people, and those same 30 people were the same 30 people for everything. And when you needed country representation, it was the same five people who are called upon. Sometimes that's the right thing but sometimes it's just tokenistic. I think in some ways, we're now seeing much more opportunity for bringing a lot more voices into conversations and I think there needs to be a lot of attention on making that happen. (Respondent 6, donor)*

While some MNH initiatives and coalitions have HFS working groups, respondents point to IAWG and the Global Health Cluster's Sexual and Reproductive Health task force as the main global groups focused specifically on SRHR in humanitarian contexts. These groups are generally perceived by respondents to operate independently from the broader MNH community.

Finally, high-level champions have demonstrated significant influence over prioritization (35). Respondents stressed their influential roles in advancing MNH on the global agenda during the MDG era including celebrities, technical advisors, and top political figures such as former UN Secretary-General Ban Ki-moon and Prime Ministers like Gordon Brown (United Kingdom), Stephen Harper (Canada), and Jens Stoltenberg (Norway). An NGO representative stated:

*We saw that [global progress] was about getting a network of people who are very passionate about maternal and newborn health but might be in more senior positions where they can bring some additional clout and attention. You're going to expect the MNCH lead to bring visibility to an issue so it's about finding some of these key players who are in more senior positions who have this passion and are maybe less expected and then asking how do we really leverage those opportunities? (Respondent 14, NGO)*

Princess Sarah Zeid of Jordan was mentioned by many respondents as the only example of a prominent, public facing individual advocating for improved MNH in HFS. While some attribute the lack of other public facing champions to a lack of political will and interest, others suggest it is due to the need to empower national champions from HFS to elevate MNH on global stages.

Respondents stated that today, progress around MNH in HFS often stems from passionate individuals working behind the scenes to bring about change within their own institutions or by actively engagement in global dialogues. The shared ENAP and EPMM milestones—including around humanitarian settings—was cited as an example of progress achieved through champion commitment.

#### 3.2.3 Interests and ideas that impact prioritization

Respondents stressed that MNH is particularly challenging given the need for clinical expertise and interventions alongside a strong health system with good governance and health financing.

Respondents assert that “everyone” is aware we will not achieve the SDG MNH targets by ignoring humanitarian and fragile settings, yet they argue the necessary solutions require decades of investments in infrastructure, training, and systems strengthening. The work remaining is seen to be the most difficult issues to address in the most difficult places and without the promise of delivering results.

One UN representative explained a growing sense of fatigue related to these investments in HFS.

*At the very beginning of a crisis, everybody's interested, and everybody wants to help. But then because some solutions are structural, they may take decades, or at least several years [to achieve]. And so there starts to be fatigue because everybody works towards the principle that there must be results. But these are very complex environments where even a small result may take so long, and a lot of resources and that fatigue only adds to the problem. And if the donor doesn't want to provide support anymore because there are so few results, there won't be the resources needed to continue making progress. So, things just fall through the cracks. (Respondent 7, UN)*

With limited resources, respondents believe donors favor “low hanging fruit” where impact is easier to achieve and sustain. This preference leads to concentrated investments in stable countries, like Malawi and Ghana, where donors anticipate “quick” wins. These countries are perceived as more likely to reach national and global MNH targets, while the same targets appear unattainable across HFS like South Sudan, Chad, and Somalia, which have some of the world's worst MNH outcomes. One donor said:

*Look at Countdown 2030, then look at how many countries are able to hit certain targets and eventually SDG targets. Some of the countries are so far off the spectrum, the sense is that it isn't attainable so it's more effective to focus on countries where success is more feasible. (Respondent 2, donor)*

Respondents noted donor hesitation to working in HFS without an exit strategy leading some to identify priority countries. According to one respondent “Donors want to have success. They want to have wins and so they look at countries where they feel they can get those wins. In humanitarian and fragile settings, there is the sense that those successes won't be possible for many years” (Respondent 2, donor). Of USAID's 25 priority countries, 10 are classified by the World Bank as fragile or conflict-affected, and 10 had 2023 humanitarian response plans yet even then, this does not mean MNH is a priority investment area (36–38). Given this, respondents argued it may be more effective to approach MNH in HFS through an equity lens rather than relying on an investment argument.

Additionally, some respondents felt that humanitarian actors rarely prioritize MNH during crises, seldom deeming it an urgent, “lifesaving” priority in comparison to food and shelter. A UN representative explained:

*Reproductive health does not come out immediately as lifesaving [in humanitarian responses]. As an SRHR community, we need to make sure we recognize and better articulate that it is lifesaving. If you don't put it as lifesaving, people are not going to prioritize it. I have been dealing with the humanitarian community for many years on these issues, and it took so many years of advocacy and working within the system to really put SRH on the map of the humanitarian community. Now it is there, but it's still not prioritized enough. The health cluster does not prioritize SRH immediately as part and parcel of what they need to do in an emergency. In Moldova, when I looked at the narrative for the first time, I was shocked. I was wondering why elements around SRH and MNH were not covered in an appeal that goes out to the international donor community. (Respondent 9, UN)*

Consequently, respondents noted there is insufficient advocacy at global and national levels to ensure MNH is prioritized in humanitarian appeals, needs overviews, and response plans and that more compelling messaging is needed. They were hopeful that a new Sexual and Reproductive Health Task Team established within the Global Health Cluster would help see these priorities more systematically addressed in all phases of humanitarian response.

### 3.4 Global policies, accountability, and coordination processes for MNH in HFS

Many global-level processes influence decisions on MNH in HFS, including research, priority setting, guideline development, and global convenings. This section outlines factors that shape these processes and influence if and how MNH in HFS is prioritized.

#### 3.2.4 The humanitarian-development divide

The humanitarian and development sectors are fundamentally different in how they operate, including in their structures, processes, and funding. Development work is traditionally funded in three- to five-year cycles while humanitarian assistance funding is often allocated in six-to-18-month spans. This separation of funding streams impacts the internal structures, processes, and coordination of MNH actors working across the nexus. Respondents noted that organizations, including donor agencies, UN agencies, and implementing organizations, often maintain separate humanitarian and development teams with limited communication and coordination. At WHO, for example, MNH is housed within the Maternal, Newborn, Child, Adolescent Health and Aging department while humanitarian response resides within the Emergencies team (39). One UN respondent indicated that the engagement between these teams does not often go beyond asking for updated guidance documents.

The division also means units often have different funding streams, procurement processes, priorities, and work plans. As a result, interviewees observed a lack of engagement between those working on MNH in stable contexts and their counterparts working in HFS. One person working for an international organization said:

*I didn't realize how separate humanitarian and development teams are. In 2018, there was a meeting that was working to bring together actors from both the development and humanitarian sides to talk about respectful [MNH] care. It was really eye opening because we were introducing colleagues from within the same organizations and agencies who had never met despite related technical focuses. Sometimes, the agencies are just so big, and people don't really have the excuse or mandate to collaborate. But it was eye opening for me to see how separate things really are and to understand what that can mean. (Respondent 12, NGO)*

This is considered problematic when there is no MNH expert on humanitarian teams, leaving a gap in technical expertise and prioritization of this issue area. At USAID for example, a respondent explained that MNH actors primarily sit within the Bureau of Global Health while humanitarian teams operate within the Bureau of Humanitarian Assistance, with minimal engagement across bureaus. They indicated that USAID's MNH team is drafting a fragile settings strategy to move away from *ad hoc* engagement but remain unclear what it may look like in practice given the agency's existing priorities and priority countries.

The division of global funding impacts country operations, particularly when the line between fragility and stability is unclear. Some respondents explained that at the national level, organizations may struggle to shift responsibilities and funds between humanitarian and development teams as crises evolve. They argued this contributes to ambiguity in addressing MNH in areas affected by protracted crises, which often bear the highest burden of maternal and newborn mortality.

Respondents expressed uncertainty regarding which entities should manage MNH in contexts perceived as too stable to be humanitarian and too fragile for development support. This division was viewed as artificial and not reflective of the operational realities on the ground. Clarifying further, a donor said:

*The division [between humanitarian and development contexts] is totally made-up, and when you're actually on the ground looking at what people are doing, it's much more a continuum and balance of trying to meet the immediate needs of people on the ground while also investing in this structures that theoretically longer term, will be able to directly meet those needs. We have chosen at the global level to kind of call that humanitarian and development but that's not realistic. They're happening much more simultaneously in most of these places, and it's mostly the same partners doing that so it's a very false division in reality. (Respondent 1, donor)*

#### 3.2.5 Advocacy and accountability toward global goals

Despite numerous MNH strategies and targets, respondents did not identify a specific mechanism for ensuring accountability for MNH investments, especially in HFS. They explained that having specific MNH targets during the MDGs, coupled with public reporting, helped to facilitate accountability. An NGO representative noted:

*I think there is definitely something to be said about that process [of tracking and sharing data]. The same can be true of how the HIV community spurred progress and accountability by establishing the 90 90 90 targets. It showed that publicly sharing data is key for accountability. (Respondent 14, NGO)*

Countdown 2030 tracks progress toward global MNH targets by analyzing data and producing reports and country profiles that aid advocacy and accountability (40). Although country profiles exist for many humanitarian and fragile contexts, one respondent highlighted concerns about the effective use of this data to hold stakeholders accountable.

According to respondents, global initiatives, and their platforms for cross-country learning, including meetings, conferences, and events, foster accountability and exert pressure on relevant actors. One researcher stated:

*Global initiatives do give some kind of accountability for the countries to have to share what they're doing, and in that process, it forces them to reflect on their current situation analysis and their priorities moving forward. Having these global initiatives and meetings also allows countries to learn from each other and draw from each other what lessons have worked or haven't worked in those contexts. (Respondent 19, research)*

Efforts to integrate humanitarian actors and perspectives have been made through establishing “humanitarian tracks” as demonstrated at the 2023 International MNH Conference and through coalitions such as ENAP and its Emergencies sub-working group. MNH reports and publications have similarly tracked progress or regression toward MNH targets with crisis-affected countries included. The advantages and limitations of this approach were noted with one person saying:

*If there is a separate humanitarian stream, it will always be thought of as an add-on. I think this is even more of an issue now because we should all be thinking about humanitarian contexts right now since it's clear we're not going to achieve the SDG targets unless we focus on the settings with the highest burden of maternal and newborn mortality. Even if we do everything else in stable settings, like everything possible to reduce mortality, we're not going to achieve the SDGs by siloing humanitarian. (Respondent 12, NGO)*

Conversely, other respondents believe that dedicated task teams or humanitarian tracks ensure humanitarian considerations are not overlooked by facilitating focused discussions among experts with shared understanding.

Global initiatives and groups like IAWG and PMNCH drive collective action to hold decision makers accountable, though their effectiveness remains unclear among respondents. Respondents further noted that while there is strong technical advocacy for MNH in HFS, there is a lack of robust public-facing advocacy, resulting in missed opportunities to attract attention and political will.

## 4 Discussion

This study examined factors influencing the prioritization of MNH in HFS on the global health agenda. Our findings indicate a notable decline in global attention and investment in MNH since the transition from the MDGs to the SDGs in 2015. The decline is attributed to a range of factors including reduced donor incentives to invest in MNH and enduring perceptions that addressing maternal and newborn deaths in HFS is complex and largely unattainable.

Development actors have increasingly stressed the localization of MNH priority-setting and decision-making. Despite this, research shows that policy elites and donors continue to exert significant influence over global and national health agendas (41). To elevate MNH in HFS on the global agenda, it is therefore essential to better understand the diverse interests of these global actors and the complex systems they operate within.

During the MDG era, the global MNH community effectively leveraged key strategies and agenda setting influence to secure substantial policy and financial commitments for MNH globally and at country levels (10, 35). This momentum has diminished during the SDG era without the equivalent high-level political leadership and attention (42).

In the competitive global health landscape, where other health priorities including disease outbreaks and pandemics, compete for attention, resource allocation often depends on the ability to generate sufficient attention for specific issues. In both humanitarian and developmental contexts, the prioritization of issues is heavily influenced by the volume and coordination of advocacy and accountability efforts. Those who can effectively convey the urgency and importance of issues are more likely to secure the funding and attention needed to drive meaningful change (43).

Our study found insufficient advocacy to keep MNH on the global development and humanitarian agendas during the SDG era. Additionally, there is a notable lack of a strong accountability mechanism to hold global and national leaders responsible for fulfilling their MNH commitments. Thus, according to respondents, elevating MNH in HFS globally requires a more focused and collaborative advocacy and accountability strategy, including establishing high-level political milestones and leveraging these to drive action and capture mainstream interest. While technical advocacy remains vital, our study emphasized that the global MNH community must also identify political champions to amplify efforts. Re-energizing PMNCH's Global Leaders Network with representatives from humanitarian settings could be a key step.

The development of country-specific MNH Acceleration Plans, introduced ahead of IMNHC 2023, has the potential to enhance attention and accountability (44). These plans aim to help national stakeholders advance MNH goals, with the global ENAP EPMM Secretariat compiling progress reports and convening a global advocacy and accountability working group. If leveraged effectively, with robust data and high-level engagement, this initiative can drive meaningful action. However, dedicated attention must be given to ensure that humanitarian and fragile settings, as well as the humanitarian actors working within them, are not an afterthought.

The study identified several other factors contributing to the perceived lack of global priority for MNH in HFS. Respondents noted that global donors increasingly prefer quick wins that show more immediate returns, viewing MNH as too challenging to address in less secure contexts. This perception hampers efforts to secure investments, leading to funding being concentrated in more stable, development contexts, while HFS experience growing funding gaps and worsening MNH outcomes (45).

This influence of donors over implementing organizations and national governments exacerbates this, as evidenced by a BRANCH Research Consortium study examining international donor influence over MNH in ten countries (34). To increase the global prioritization of MNH in HFS, global actors must shift the narrative, craft compelling messages, and advocate for investments in HFS. Developing shared messaging and identifying common interests among donors, development, and humanitarian actors can provide entry points for effective advocacy and investment. Additionally, innovative MNH funding mechanisms that bridge the humanitarian-development nexus could enable longer-term investments in humanitarian settings and address barriers related to disparate funding sources, timelines, and processes.

While the study recognizes efforts to include HFS in global MNH initiatives, it also highlights that MNH in HFS is often seen as a secondary or separate issue. Notably, a significant number of EPMM focus countries (7 out of 19), Quality of Care network countries (10 out of 22), ENAP priority countries (13 out of 36), Global Financing Facility partner countries (14 out of 36), and first round countries developing MNH Acceleration Plans (10 out of 29), have active UN humanitarian appeals as outlined in Table 1 (3). Despite this, these initiatives often lack a dedicated focus on HFS.

**Table 1:** Humanitarian settings targeted by global MNH initiatives.

Global MNH initiative	Countries with active UN humanitarian appeal
Quality of Care network member countries	Cameroon, Chad, DRC, Ethiopia, Mozambique, Myanmar, Niger, Nigeria, South Sudan, Yemen
ENAP priority countries	Afghanistan, Central African Republic (CAR), Chad, DRC, Ethiopia, Mali, Mozambique, Niger, Nigeria, Somalia, South Sudan, Sudan, Yemen
EPMM priority countries	Afghanistan, Cameroon, Chad, Ethiopia, Nigeria, Somalia, Sudan
MNH Acceleration plans (ENAP/EPMM) – first round countries	Burkina Faso, CAR, Ethiopia, Guatemala, Haiti, Mali, Mozambique, Nigeria, Somalia, Yemen
GFF partner countries	Afghanistan, Burkina Faso, Cameroon, CAR, Chad, DRC, Ethiopia, Guatemala, Mali, Mozambique, Myanmar, Niger, Nigeria, Somalia

Even more, the study points to a belief that humanitarian settings fall outside the scope of development investments. Closing this gap requires adopting an equity lens, emphasizing that global and regional targets cannot be met without addressing the needs of these contexts. Ensuring the inclusion of humanitarian actors in global decision-making forums is imperative and may involve designating quotas for humanitarian actors in all global MNH convenings. Agencies with expertise in both development and humanitarian settings should proactively exchange priorities and insights to avoid duplication and address coverage gaps. In cases where humanitarian teams lack an MNH focal point, regular consultations are essential to ensure MNH expertise is integrated into humanitarian response activities.

Efforts to enhance coordination have been made through initiatives like the Core Group's Humanitarian-Development Nexus Collaboration Hub and Momentum Integrated Health Resilience's (MIHR) conceptual framework for health programming in the Humanitarian-Development Nexus (46, 47). As these and similar efforts continue, documenting learnings and success stories will be crucial to identifying the most effective models and frameworks for bridging the gap between humanitarian and development actors.

Looking ahead, our study highlights that the MNH sector is at a critical juncture. Recent initiatives such as the 2023 International MNH Conference and the 90/90/80/80 targets, are key to reenergizing the MNH community and catalyzing action (17). However, if global priorities do not shift to focus more on HFS, we risk missing global MNH targets and seeing continued stagnation in reducing preventable deaths.

Although this paper chose to focus on the perspectives of global actors, we did not intentionally exclude stakeholders in the Global South. Despite ongoing efforts to decolonize global health, a report published in 2020 outlines data revealing that a substantial majority (85%) of global health organizations are based in North America, Europe, and Oceania, with two-thirds headquartered in Switzerland, the UK, and the USA. Moreover, leadership roles within these organizations are predominantly occupied by older males from high-income countries (15). Therefore, in our effort to identify individuals engaged in global MNH agenda-setting and decision-making spaces, we saw a clear gap in Global South representation, underscoring the need for proactive advocacy to promote the diversity of voices and perspectives when defining global MNH policy, practice, and financing priorities.

While acknowledging the potential for bias and homogeneity in our sampling approach, we sought to authentically capture the perspectives and experiences of influential actors within this specialized field and to accurately reflect their roles and positions within the global health system.

At the same time, we acknowledge the importance of understanding the synergies and tensions between national and global levels, particularly how global actions and priorities manifest at the national and subnational levels. Capturing the perspectives of those directly involved in MNH in affected settings would be essential for this understanding. While the EQUAL research consortium (under which this study was conducted), is studying MNH prioritization in eastern DRC, northeast Nigeria, Somalia, and South Sudan, future research should further explore how global power dynamics influence country-level decisions and document the perspectives of national actors on these dynamics.

## 5 Limitations

The study encountered numerous limitations. Our search terms did not explicitly include “child health” or “gender”, which are closely associated with newborn and maternal health, respectively. This oversight may have resulted in missing relevant literature that could have enriched our understanding of global MNH priorities. The study team found a dearth of evidence exploring the complex global systems, structures, and processes that influence the prioritization of MNH in HFS on the global health agenda. Furthermore, the study faced challenges related to limited availability of individuals working specifically on MNH in humanitarian settings at the global level. Some individuals identified during sampling were excluded due to their direct association with the EQUAL research consortium or the organization of the authors. Time constraints during interviews, limited to roughly 60 min due to respondent availability, may have impacted the depth and nuance of the topics discussed. Lastly, the focus of our study and sampling strategy resulted in almost all interviewees being based in the Global North. While efforts to decentralize the headquarters of organizations like UNFPA to Global South locations will help support better representation in the future, our study could have explored a more inclusive approach to sampling.

## 6 Conclusion

This study adds to the limited evidence on the global prioritization of MNH in HFS and offers findings that may contribute to ongoing international discourse on this topic. The study identifies several barriers that undermine prioritization including a fragmented global policy community across development and humanitarian actors, a lack of political champions, the absence of a strong civil society focused specifically on MNH in HFS, and prevailing perceptions that intervention in these settings is particularly difficult due to insecurity and weak systems. Nevertheless, the study highlights key strategies and initiatives that can be strengthened to elevate MNH in HFS on the global agenda. As crises, conflict, and climate change exacerbate fragility in more regions, global stakeholders must prioritize addressing health impacts, including for MNH. Moving forward requires renewed leadership, targeted investment, and robust advocacy. By leveraging new opportunities and overcoming identified barriers, meaningful progress can be made to reduce mortality and achieve global MNH targets.

## Data Availability

The raw data supporting the conclusions of this article will be made available by the authors, without undue reservation.

## Footnotes

^1^ *In 2014, 194 Member States of the Sixty-seventh World Health Assembly endorsed the Every Newborn Action Plan (Resolution WHA 67.10)*
