# Supplementary material for: Stakeholder perspectives on factors that influence global prioritization for MNH in humanitarian settings
Source: Front Glob Womens Health. 2024 Aug 26;5:1364603. doi: 10.3389/fgwh.2024.1364603 (PMC11381243; doi:10.3389/fgwh.2024.1364603)
Supplement: Supplementary file 1 [file Table1.pdf]

Supplementary 1: Global MNH Content

The following represents a non-comprehensive list of global MNH documents identified during the desk review. These documents were all produced/updated after 2015 including guidelines, reports, strategies, and targets. It offers a brief content summary and assesses the extent to which humanitarian settings are addressed. This analysis involved source identification through a desk review and snowballing and a keyword search for “humanitarian” “fragile” “crisis” “conflict” and “emergency” to determine the extent to which humanitarian settings were included.

**Global MNH Strategies**		
**Name of document**	**General overview**	**Humanitarian considerations**
Global Strategy for Women’s, Children’s and Adolescents’ Health (2016-2030)(1)	Presents a roadmap to ending all preventable deaths of women, children and adolescents within a generation and ensuring their well-being. Targets are aligned to the SDGs.	Acknowledges that SDGs cannot be achieved without attention to humanitarian and fragile settings.<br><br>Chapter 7 focuses on humanitarian and fragile settings with emphasis on building resilience and offers recommendations including addressing gaps of working across the nexus, integrating emergency preparedness and response into national health plans. The 2022 progress report focuses on climate, covid, and conflict.
Every Newborn Action plan (2)	A road map of strategic actions for ending preventable newborn mortality and stillbirth and contributing to reducing maternal mortality and morbidity. It was endorsed by 194 Member States at the World Health Assembly in 2014.	Acknowledges the high burden of mortality in humanitarian contexts. Calls for newborn health indicators to be added and tracked including in places with complex emergencies.
Strategies for ending preventable maternal mortality (EPMM) (3)	Outlines broad strategies for strengthening maternal health programs.	Milestone 7 urges all countries to have a preparedness and response plan including efforts to promote MNH, prevent stillbirths and have a coordinated mechanism in place for its implementation.
**Global MNH targets**		
**Name of document**	**General overview**	**Humanitarian considerations**

Millennium Development Goals (4)	The UN Millennium Development Goals (MDGs) were a set of 8 goals that UN Member States agreed to try to achieve by the year 2015. Goal 5 aimed "To Improve Maternal Health" with the following sub-targets:<br>• Target 5.A: Reduce by three quarters, between 1990 and 2015, the maternal mortality ratio<br>• Target 5.B: Achieve, by 2015, universal access to reproductive health	There is no specific reference to humanitarian settings in the MDG targets.
Sustainable Development Goals (5)	17 goals intended to be achieved by 2030. SDG 3 is "ensure healthy lives and promote well-being for all at all ages" includes MNH targets:<br>● Target 3.1: "by 2030, reduce the global maternal mortality ratio to less than 70 per 100,000 live births"<br>● Target 3.2: "by 2030, end preventable deaths of newborns and children under 5 years of age, with all countries aiming to reduce neonatal mortality to at least as low as 12 per 1,000 live births and under-5 mortality to at least as low as 25 per 1,000 live births."	There is no specific mention of /or targets set for humanitarian contexts. The intention was for the goals/targets to apply to all countries.
Ending Preventable Maternal Mortality (3)	Ending Preventable Maternal Mortality coverage targets & milestones to achieve by 2025:<br>● 90% pregnant women to attend four or more antenatal care visits (increasing to eight visits by 2030)<br>● 90% births to be attended by skilled health personnel<br>● 80% women who have just given birth to access postnatal care within two days of delivery<br>● 60% of the population to have access to emergency obstetric care within two hours of travel time<br><br>Global target: By 2030, all countries should reduce maternal mortality ratio (MMR) by at least two-thirds of their 2010 baseline level. The average global target is an MMR of less than 70 maternal deaths per 100 000 live births by 2030.<br><br>National target: By 2030, no country should have an MMR greater than 140, a number twice the global target.	See above regarding response and resilience milestone/target

Every Newborn Action Plan (2)	• Less than 12 newborn deaths per 1000 live births<br>• Less than 12 stillbirths per 1000 total births by 2030.<br>• 80% of countries have national implementation plans that are being implemented in at least half of the country with an appropriate number of functional level 2 inpatient units linked to level 1 units to care for small and sick networks with family-centered care.	Aligns with the Ending Preventable Maternal Mortality coverage targets and milestones including around response and resilience.
Every Newborn Action Plan / Ending Preventable Maternal Mortality global targets	• 90% pregnant women receiving at least four antenatal care contacts.<br>• Skilled health personnel attending 90% births.<br>• 80% of new mothers and babies receiving postnatal care within two days of birth.<br>• 80% of districts across countries having access to emergency obstetric services and small and sick newborn care.	No specific reference to humanitarian contexts but it is intended to apply to all contexts.
**WHO Guidelines (non-comprehensive)**		
**Name of document**	**General overview**	**Humanitarian considerations**

**Maternal Health**		
Home-based records for maternal, newborn, and child health (6)	Recommends the use of home-based records as a complement to facility-based records to improve care-seeking behaviors, male involvement, maternal and child home care practices, infant and child feeding, and communication between health providers and women/caregivers.	Acknowledges that in fragile settings home-based records may be of greater value than in more developed settings and health systems.
Antenatal care for a positive pregnancy experience (7)	Global, evidence-informed recommendations on routine antenatal care occurring in any healthcare or community setting.	No mention of humanitarian settings.
Recommendations on maternal health (8)	Responds to key questions including what health interventions should be delivered during pregnancy, childbirth and the postnatal period and what health behaviors should the women practice (or not practice) during these periods.	No mention of humanitarian considerations/settings.
Maternal and perinatal death and surveillance and response- Guidance and materials (9)	A roadmap for conducting MPDSR in clinical and policy settings. Offers practical step by step guidance to assess the burden of maternal deaths, stillbirths and neonatal deaths.	Includes a module for MPDSR in humanitarian and fragile settings

**Labor and delivery**		
Intrapartum care for a positive childbirth experience (10)	Guidelines for essential intrapartum care including new and existing WHO recommendations that, when delivered as a package, will ensure good-quality and evidence-based care irrespective of the setting or level of health care.	Acknowledge the variations that exist globally as to the level of available health services within and between countries.
Recommendations for non-clinical interventions to reduce unnecessary cesarean sections (11)	A guideline on non-clinical interventions to reduce unnecessary cesarean sections. It incorporates the views, fears and beliefs of both women and health professionals.	No reference
Recommendations on uterotonics for the prevention of postpartum hemorrhage (12)	Guidelines/recommendations related to the use of uterotonics during the third stage of labor for the prevention of PPH, regardless of birth setting (hospital or community setting).	No reference
Recommendation on advance misoprostol distribution to pregnant women for prevention of postpartum hemorrhage (13)	Indicates in settings where women give birth outside of a health facility and in the absence of skilled health personnel, a strategy of antenatal distribution of misoprostol to pregnant women for self-administration is recommended for prevention of postpartum hemorrhage.	Encourages the adaptation, integration, and alignment of these recommendations with other response strategies. Suggests context-specific tools and toolkits to support its implementation in humanitarian emergencies.
Labour care guide: user's manual (14)	A tool to support good-quality, evidence-based, respectful care during labor and childbirth, irrespective of the setting or level of health care.	No reference.

**Perinatal/postnatal care**		
Iron supplementation in postpartum women (15)	Guideline to support informed decisions on appropriate nutrition actions related to iron supplementation.	No reference
Recommendations on maternal and newborn care for a positive postnatal experience (16)	Aims to improve the quality of essential, routine postnatal care for women and newborns and consolidates new and existing recommendations on routine PNC for women and newborns receiving facility- or community-based postnatal care in any resource setting.	Encourages adaptation in humanitarian settings and integration and alignment with other response strategies.
Guide for integration of perinatal mental health in maternal and child health services (17)	Outlines an evidence-informed approach describing how program managers, health service administrators and policy-makers responsible of planning and managing maternal and child health services can develop and sustain high-quality, integrated mental health services for women during the perinatal period.	Includes a section on humanitarian settings including specific considerations.

**Newborn health**		
Recommendations on newborn health (18)	Responds to questions on health interventions that should be applied for newborns and young infants less than 2 months of age and when.	No reference
Standards for improving the quality of care for small and sick newborns in health facilities (19)	Aims to define, standardize, and mainstream inpatient care of small and sick newborns and to guide countries in caring for this vulnerable population and support the quality of care of newborns in the context of universal health coverage.	Acknowledges humanitarian settings including the need for special considerations to the specific psychosocial and practical needs of small and sick newborns and their careers. Emphasizes inclusion of small and sick newborns in preparedness, response and recovery plans. Outlines recommendations in line with the Newborn health in humanitarian settings: field guide
Recommendations for care of the preterm or low-birth-weight infant (20)	Informs the development of national and subnational health policies, clinical protocols and programmatic guides related to the planning and management of maternal, newborn and child health services, especially for preterm and low-birth-weight babies.	Encourage integration and alignment with response strategies and emphasizes that additional considerations should be made for emergency settings.
Survive and Thrive: transforming care for every small and sick newborn (21)	Maps a pathway towards 2030 building upon epidemiology, historical trends, lessons learnt and evidence-based interventions.	Includes a chapter on newborn health in humanitarian settings but refers to other documents for specific recommendations.

**Quality of Care**<br><br>Standards for improving quality of maternal and newborn care in health facilities (19)<br><br>Quality of Care in Humanitarian Settings - global health cluster guide (22)	Define what is required to achieve high-quality care around the time of childbirth. Addresses clinical guidelines, standards of care, effective interventions, measures of quality of care, relevant research and capacity-building.<br><br>Defines the scope and minimum issues that must be considered when addressing quality of care in humanitarian settings and should be referred to where the Cluster system has been established. It is relevant for both acute and protracted crises.	No reference<br><br>Focused on humanitarian settings and developed in complement SRHR guidance. Does not explicitly mention MNH.
**Community Health**<br><br>Guideline on health policy and systems support to optimize community health worker programs (23)	Aims to assist national governments and national and international partners to improve the design, implementation, performance, and evaluation of CHW programs.	No reference
**Health workforce**<br><br>Human resource strategies to improve newborn care in health facilities in low and middle income countries (24)	A framework for countries to transform their policies on human resources for health and provide health workers with the knowledge and technical and behavioral skills necessary for high-quality care by 2030 to ensure newborns survive and thrive.	Acknowledges human resource gaps and needs in humanitarian settings but does not include specific guidance or recommendations.

**Miscellaneous**<br><br>Guideline on self-care interventions for health and well-being (25)	Provides evidence-based normative guidance that will support individuals, communities and countries with quality health services and self-care interventions based on primary healthcare strategies, comprehensive and essential service packages. It includes a section and recommendation on MNH.	Includes key considerations and recommendations for humanitarian settings.
MNH in Humanitarian Settings Resources and roadmaps (non-comprehensive)		
**Name of document**	**General overview**	**Humanitarian considerations**
Minimum Initial Service Package (MISP) (26)	Outlines the series of crucial, lifesaving activities required to respond to the SRH needs of affected populations at the onset of a humanitarian crisis. MNH is one chapter/module.	The focus is explicitly on humanitarian settings.
Roadmap to Accelerate Progress for Every Newborn in Humanitarian Settings 2020 – 2024 (27)	Designed to further the global commitment to improve the health of the most vulnerable mothers and newborns who live in humanitarian settings. It is based on global evidence-based guidance and lessons learned from country-level implementation.	Focused on humanitarian settings.
Newborn Health in Humanitarian Settings: Field Guide (28)	Designed as an enhancement to national strategies and programs aimed at improving the lives of newborns and their mothers, and to strategies such as the Every Newborn Action Plan.	Focused on humanitarian settings
Inter-Agency Field Manual on RH in Crises (23)	The authoritative source for SRH in crises guided by global norms, integrating evidence and examples to apply and adapt sexual and reproductive health (SRH) and human rights standards in various crises.	Focused on humanitarian settings and has a chapter on MNH.

Global Reports		
**Name of document**	**General Overview**	**Humanitarian considerations**
Annual SDG Progress report (29)	Issued by the UN Secretary General with data from UN member states. The report is coupled with the Global SDG Indicators Database which includes global, regional and country data and metadata on the official SDG indicators including MNH indicators (39). In theory this report provides the best reflection of globally comparable data toward the SDG target.	Countries impacted by humanitarian crises and fragility are included, yet data captured in these contexts is often weak.
Survive & thrive – transforming care for every small and sick newborns (21)	Outlines the global challenges faced by small and sick newborns emphasizing progress made and summarizes what can be done to transform inpatient care for this vulnerable population.	Recognizes that to achieve the SDG MNH targets, the global community must work to reach the most vulnerable populations in these areas, especially in HFS.
Ending preventable newborn deaths and still births (30)	A roadmap to end preventable newborn mortality, reduce disability and end preventable stillbirths by 2030. The document sets out new global, national, and subnational targets and key actions that can be taken in partnership to accelerate progress.	Acknowledges the burden in humanitarian settings.
State of the World's Children (31)	A flagship report examining key issues affecting children.	Includes data for MNH indicators for countries impacted by humanitarian crises. There is a section on humanitarian crises, but it does not reference MNH.
State of the World's Midwives (32)	Document the whole world's Sexual, Reproductive, Maternal, Newborn and Adolescent Health (SRMNAH) workforce, with a focus on midwives. It calls for urgent investment in midwives to enable them to fulfill their potential to contribute towards UHC and the SDG agenda.	There is a "humanitarian" focused supplement that addresses MNH needs in humanitarian contexts and the role of midwives in meeting those needs.
A Neglected Tragedy: The global burden of stillbirths (33)	The first stillbirth report by the UN Inter-Agency Group for Child Mortality Estimation (UN-IGME), helping to improve the availability of data globally and driving political and public recognition of the issue.	Acknowledges that the greatest burden of stillbirths is in fragile and conflict-affected settings; shows data for burden including % increase between 2000-2019 including for countries affected by crisis.

Every Newborn Progress Report (34)	Reports on progress toward identified national and sub-national Every Newborn Action Plan targets and milestones.	Includes a section on newborn health in humanitarian settings with recognition that these settings account for the highest burden of mortality; data on if/where newborn health is included in preparedness and response plans.
PMNCH accountability reports (35)	Used to track commitments annually as part of the EWEC and H6 work plan.	Includes analyses of commitments in humanitarian and fragile settings.
Trends in maternal mortality 2000 to 2020 (36)	Presents internationally comparable global, regional and country-level estimates and trends for maternal mortality between 2000 and 2020.	Acknowledges that trends have stagnated, and, in some cases, outcomes have gotten worse in humanitarian, fragile, and conflict-affected contexts.
Born too Soon 2023 Report (37)	Sets an ambitious agenda to reduce the burden of preterm birth. Intended to inspire and support country-led action: politicians, policymakers and leaders of all stakeholder groups are its primary audience.	Acknowledges the high burden in humanitarian settings; includes a section on emergencies and a profile on Yemen.
Improving maternal and newborn health and survival and reducing stillbirth - Progress report 2023 (38)	Presents key findings and priority actions around maternal mortality, stillbirths, and neonatal mortality data as well as new data on country progress towards the ENAP-EPMM coverage targets and milestones.	Acknowledge the high burden in humanitarian settings and calls for use of context specific indicators for humanitarian and fragile settings.

1. Child EWE. The Global Strategy for Women's, Children's and Adolescent's Health (2016-2030). New York: Every Woman Every Child; 2105.
2. WHO, UNICEF. Every Newborn: An Action Plan to End Preventable Deaths. Geneva: World Health Organization; 2014.
3. Strategies toward ending preventable maternal mortality (EPMM). Geneva: World Health Organization; 2015.
4. Nations U. We Can End Poverty: Millenium Development Goals and Beyond 2015: UN; 1990 [Available from: https://www.un.org/millenniumgoals/maternal.shtml.
5. Sustainable Development Goals: United Nations; 2015 [Available from: https://sdgs.un.org/goals.
6. WHO recommendations on home-based records for maternal, newborn and child health. Geneva: World Health Organization; 2018. Contract No.: CC BY-NC-SA 3.0 IGO.
7. WHO recommendations on antenatal care for a positive pregnancy experience. Geneva: World Health Organization; 2016. Contract No.: CC BY-NC-SA 3.0 IGO.
8. WHO recommendations on maternal health: guidelines approved by the WHO Guidelines Review Committee. Geneva: World Health Organization; 2017. Contract No.: CC BY-NC-SA 3.0 IGO.
9. Maternal and perinatal death and surveillance and response. Geneva: World Health Organization; 2021. Contract No.: Licence: CC BY-NC-SA 3.0 IGO.
10. WHO recommendations: intrapartum care for a positive childbirth experience. Geneva: World Health Organization; 2018. Contract No.: Licence: CC BY-NC-SA 3.0 IGO.
11. WHO recommendations: non-clinical interventions to reduce unnecessary caesarean sections. Geneva: World Health Organization; 2018. Contract No.: Licence: CC BY-NC-SA 3.0 IGO.
12. WHO recommendations Uterotonics for the prevention of postpartum haemorrhage. Geneva: World Health Organization; 2018. Contract No.: Licence: CC BY-NC-SA 3.0 IGO.
13. WHO recommendation on advance misoprostol distribution to pregnant women for prevention of postpartum haemorrhage. Geneva: World Health Organization; 2020. Contract No.: Licence: CC BY-NC-SA 3.0 IGO.
14. WHO labour care guide: user’s manual. Geneva: World Health Organization; 2021. Contract No.: CC BY-NC-SA 3.0 IGO.
15. Guideline: Iron supplementation in postpartum women. Geneva: World Health Organization; 2016.
16. WHO recommendations on maternal and newborn care for a positive postnatal experience. Geneva: World Health Organization; 2022. Contract No.: Licence: CC BY-NC-SA 3.0 IGO.
17. WHO guide for integration of perinatal mental health in maternal and child health services. Geneva: World Health Organization; 2022. Contract No.: Licence: CC BY-NC-SA 3.0 IGO.
18. WHO recommendations on newborn health: guidelines approved by the WHO Guidelines Review Committee. Geneva: World Health Organization; 2017. Contract No.: (WHO/MCA/17.07). Licence: CC BY-NC-SA 3.0 IGO.
19. Standards for improving the quality of care for small and sick newborns in health facilities. Geneva: World Health Organization; 2020. Contract No.: Licence: CC BY-NC-SA 3.0 IGO.

20. WHO recommendations for care of the preterm or low-birth-weight infant. Geneva: World Health Organization; 2022. Contract No.: Licence: CC BY-NC-SA 3.0 IGO.
21. Survive and thrive: transforming care for every small and sick newborn. Geneva: World Health Organization; 2019. Contract No.: Licence: CC BY-NC-SA 3.0 IGO.
22. Quality of Care in Humanitarian Settings. Health Cluster; 2020.
23. WHO guideline on health policy and system support to optimize community health worker programmes. Geneva: World Health Organization; 2018. Contract No.: Licence: CC BY-NC-SA 3.0 IGO.
24. Human resource strategies to improve newborn care in health facilities in low- and middle- income countries. Geneva; 2020. Contract No.: Licence: CC BY-NC-SA 3.0 IGO.
25. WHO guideline on self-care interventions for health and well-being. Geneva: World Health Organization; 2022. Contract No.: Licence: CC BY-NC-SA 3.0 IGO.
26. (IAWG) I-AWGoRHiC. Inter-Agency Field Mannual on Reproductive Health in Humanitarian Settings. Minimum Initial Service Package (MISP)2018.
27. Children St, WHO, UNICEF, UNHCR. Roadmap to Accelerate Progress for Every Newborn in Humanitarian Settings 2020 – 2024. Washington, DC: Save the Children; 2020.
28. UNICEF, Children St. Newborn Health in Humanitarian Settings Field Guide. New York: UNICEF; 2018.
29. The SDG Goals Report. New York: United Nations; 2022.
30. Ending Preventable Maternal and Newborn Deaths and Stillbirths by 2030. Geneva: World Health Organization; 2020.
31. State of the World's Children. New York: UNICEF; 2021.
32. State of the World's Midwife. New York: UNFPA; 2021.
33. A Neglected Tragedy: The global burden of stillbirths. New York: UNICEF; 2020.
34. Every Newborn Progress Report. New York: World Health Organization and the United Nations Children’s Fund (UNICEF),; 2019. Contract No.: Licence: CC BY-NC-SA 3.0 IGO.
35. PMNCH Annual Report. Geneva: PMNCH; 2022.
36. Trends in maternal mortality 2000 to 2020: estimates by WHO, UNICEF, UNFPA, World Bank Group and UNDESA/Population Division. Geneva: World Health Organization; 2023. Report No.: Licence: CC BY-NC-SA 3.0 IGO.
37. Born too soon: decade of action on preterm birth. Geneva: World Health Organization; 2023. Contract No.: Licence: CC BY-NC-SA 3.0 IGO.
38. Improving maternal and newborn health and survival and reducing stillbirth: progress report 2023. Geneva: World Health Organization; 2023. Contract No.: Licence: CC BY-NC-SA 3.0 IGO.
39. SDG Indicators Database New York: United Nations Department of Economic and Social Affairs; [Available from: https://unstats.un.org/sdgs/dataportal.
