# Supplementary figures and images for: Stakeholder perspectives on factors that influence global prioritization for MNH in humanitarian settings
Source: Front Glob Womens Health. 2024 Aug 26;5:1364603. doi: 10.3389/fgwh.2024.1364603 (PMC11381243; doi:10.3389/fgwh.2024.1364603)

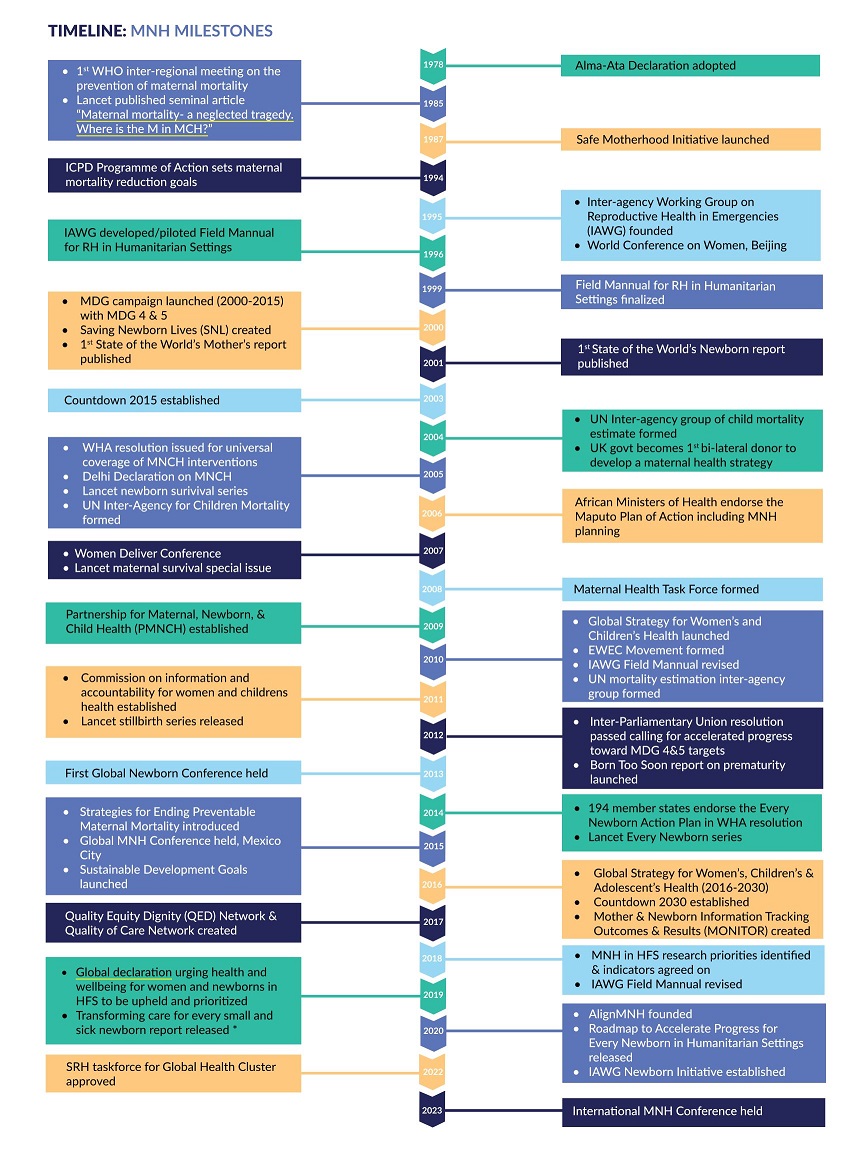

Supplement: Supplementary file 2 [file Image1.jpeg]
